# The relationship between corticospinal excitability and behavioural measures of movement imagery ability

**DOI:** 10.1093/braincomms/fcag013

**Published:** 2026-01-16

**Authors:** Marcos Moreno-Verdú, Laurine Boidequin, Baptiste M Waltzing, Elise E Van Caenegem, Charlène Truong, Gautier Hamoline, Robert M Hardwick

**Affiliations:** Brain, Action, and Skill Laboratory (BAS-Lab), Institute of Neuroscience (Cognition and Systems Division), UCLouvain, 1200 Woluwe-Saint-Lambert, Brussels, Belgium; Brain, Action, and Skill Laboratory (BAS-Lab), Institute of Neuroscience (Cognition and Systems Division), UCLouvain, 1200 Woluwe-Saint-Lambert, Brussels, Belgium; Brain, Action, and Skill Laboratory (BAS-Lab), Institute of Neuroscience (Cognition and Systems Division), UCLouvain, 1200 Woluwe-Saint-Lambert, Brussels, Belgium; Brain, Action, and Skill Laboratory (BAS-Lab), Institute of Neuroscience (Cognition and Systems Division), UCLouvain, 1200 Woluwe-Saint-Lambert, Brussels, Belgium; Brain, Action, and Skill Laboratory (BAS-Lab), Institute of Neuroscience (Cognition and Systems Division), UCLouvain, 1200 Woluwe-Saint-Lambert, Brussels, Belgium; Brain, Action, and Skill Laboratory (BAS-Lab), Institute of Neuroscience (Cognition and Systems Division), UCLouvain, 1200 Woluwe-Saint-Lambert, Brussels, Belgium; Brain, Action, and Skill Laboratory (BAS-Lab), Institute of Neuroscience (Cognition and Systems Division), UCLouvain, 1200 Woluwe-Saint-Lambert, Brussels, Belgium

**Keywords:** movement imagery, motor imagery, corticospinal excitability, TMS, SICI

## Abstract

Imagining a movement without executing it has measurable effects on physical performance, learning, and rehabilitation. However, these effects rely on our ability to imagine performing actions, a complex, covert skill that is difficult to quantify. While movement imagery ability can be assessed by behavioural methods or measuring its neural correlates, the relationship between these measures is uncertain. This Registered Report will determine the association between three key behavioural processes during movement imagery – generation, maintenance and manipulation – and well-established neurophysiological measures of corticospinal excitability and intracortical inhibition during imagery, obtained via Transcranial Magnetic Stimulation. A behavioural battery including a questionnaire, a ‘mental chronometry’ task, and a hand rotation task will be collected alongside the amplitude of Motor Evoked Potentials and the change in Short Interval Cortical Inhibition during imagery. Bayesian correlations will assess the association between these measures to provide a comprehensive evaluation of the neuro-behavioural correlates of movement imagery.

## Teaser

Moreno-Verdú *et al*. report that this Registered Report will determine how behavioural processes of movement imagery – generation, maintenance and manipulation – relate to corticospinal excitability and intracortical inhibition measured with Transcranial Magnetic Stimulation. Bayesian correlations will provide a comprehensive evaluation of the neuro-behavioural links underlying movement imagery.

## Graphical Abstract

**Graphical Abstract:**
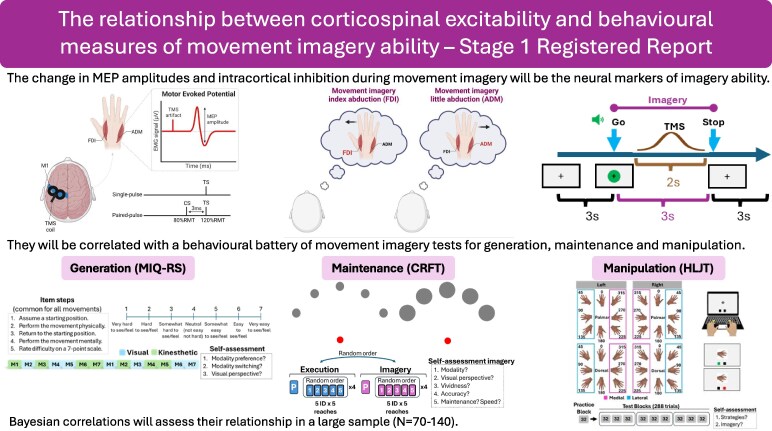

## Introduction

Our ability to imagine an action without executing it is of longstanding and widespread interest to scientists, clinicians, athletes, and philosophers. Our capacity to perform ‘movement imagery’^1^ is made possible through mental processes combining neuromotor control, perception, and higher-order cognitive functions.^2^ Movement imagery is particularly relevant as a complementary method to enhance motor learning – especially when physical practice is restricted (e.g. in rehabilitation) – as there is evidence that coupling mental and physical practice leads to greater improvements in performance in a variety of motor skills.^3,4^ This has been observed across different stages of learning^5,6^ and for several motor domains.^7,8^ Movement imagery training, therefore, has applications across a broad and diverse range of fields, including sports and rehabilitation.^9-11^

It has long been argued that the capacity to perform movement imagery (i.e. ‘movement imagery ability’) varies across individuals,^12,13^ which should in turn affect an individual’s ability to benefit from using movement imagery training.^14^ This has led to the development of ‘movement imagery ability assessments’, which aim to determine performance in movement imagery tasks.^15,16^ Much research has been conducted into finding *valid* behavioural measures of imagery ability. So far, measures aiming to assess three different ‘processes’ of movement imagery – generation, maintenance and manipulation―have been proposed.^17,18^ Generation – the ability to bring a high-quality sensory representation to the mind’s eye – is commonly assessed through self-report questionnaires.^16^ Technically, these questionnaires evaluate the intensity (also referred to as vividness) with which the individual perceives the imagined movement, or the perceived ease of creating the mental representation. Classically, two sensory modalities are considered in imagery questionnaires, namely visual (seeing the movement from a first- or third-person perspective) and kinaesthetic (feeling the movement, usually from a first-person perspective).^16^ Maintenance – the ability to sustain the representation over time and with temporal precision – is usually assessed through mental chronometry paradigms.^19^ These paradigms evaluate the temporal relationship of the imagined movement with its physical counterpart; the closer the times, the better the ability to precisely maintain movement imagery until the action is completed. Manipulation – the ability to dynamically transform the content and/or characteristics of the mental representation – is often assessed through mental rotation tasks. The most used paradigm is the Hand Laterality Judgement Task (HLJT), in which participants decide whether rotated images of hands belong to the right or left side of the body, with measures of accuracy and reaction time typically employed to determine performance.^20-22^

In spite of the ubiquitous use of imagery ability assessments in both research and applied contexts, their biological validity is still debated.^23^ There is an ample body of neuroimaging research suggesting movement imagery activates a brain network largely overlapping with the classical sensory-motor (or action-related) network.^24,25^ Furthermore, movement imagery produces an increase in corticospinal excitability as assessed through Motor Evoked Potentials (MEPs) in response to single-pulse Transcranial Magnetic Stimulation (TMS).^26,27^ There is also converging evidence that movement imagery produces intracortical ‘disinhibition’ in the motor cortex assessed through paired-pulse TMS (i.e. lower inhibition in a Short Interval Intracortical Inhibition (SICI) protocol during imagery compared to rest).^28-32^ Nonetheless, this latter effect can vary depending on methodological aspects such as direction of TMS-induced currents, conditioning stimulus intensity, or the nature of the imagined movement.^33-36^ Based on the above evidence, movement imagery ability could be assessed by measuring the strength with which an individual produces corticospinal facilitation or intracortical disinhibition during imagery, illustrating the degree of recruitment of brain regions within the action (sensory-motor) network.^27^ In other words, larger increases in corticospinal facilitation, or greater reductions of intracortical inhibition, may represent a better ability to activate the action network during movement imagery. However, a key question remains as to whether individuals exhibiting *higher* ability to perform movement imagery according to behavioural measures would demonstrate *stronger* neurophysiological effects of movement imagery. This is necessary as TMS-derived measures may not always be feasible or possible to collect from a given individual, hence behavioural measures may be used preferentially in this scenario.

Prior evidence on the relationship between corticospinal facilitation (increase in MEPs) and behavioural measures of movement imagery is conflicting, and studies examining this question have generally been statistically underpowered. While some analyses indicate moderate relationships (i.e. individuals with ‘higher’ ability also show greater corticospinal facilitation), other results show no *statistically significant* relationships between these variables.^37-39^ The strength of the association partially depends on which process of movement imagery ability is being considered (generation or manipulation have been specifically investigated so far), as well as which concrete test is used. Studies have shown negligible or weak-to-moderate correlations with the HLJT (as a measure of manipulation) as well as with the kinaesthetic subscales of questionnaires (as measures of generation) or trial-to-trial vividness, independently.^37-39^ No studies have assessed the relationship with measures of maintenance (mental chronometry), but a combined index (using a questionnaire, mental chronometry and physiological tests like skin conductance) weakly correlated with corticospinal facilitation.^27^ However, intracortical disinhibition in the motor cortex during movement imagery was not different between ‘good’ and ‘poor’ imagers according to this measure.^27^ Regardless, these prior works all had very limited sample sizes (*n* < 25 participants) that were markedly underpowered for classical correlation analyses under traditional frequentist Null Hypothesis Significance Testing (NHST). Consequently, the actual existence and strength of the relationship between behavioural and neurophysiological measures of movement imagery remains unclear, highlighting the need for this question to be examined with suitably powered studies.

This Registered Report will therefore elucidate the relationship between behavioural and neurophysiological measurements of movement imagery. Imagery ability scores on a comprehensive battery of behavioural tasks will be correlated with the change in MEP amplitude associated with movement imagery in the largest sample so far. We predict (Hypothesis 1 – see Table 1) that individuals showing ‘higher’ imagery ability according to behavioural assessments will exhibit greater corticospinal facilitation during movement imagery (i.e. a correlation will be observed in the expected direction). We will also measure the strength of intracortical ‘disinhibition’ produced during movement imagery, predicting (Hypothesis 2) that individuals showing higher imagery ability will exhibit stronger disinhibition. We will use Bayesian correlations with pre-defined stopping criteria for evidence in favour of the null or alternative hypotheses to ascertain whether MEPs and imagery ability scores are associated. This study will therefore comprehensively address the fundamental question of brain-behaviour relationships during imagery, advancing our understanding of movement imagery ability and its evaluation, which has potentially wide-ranging applications for both fundamental and applied situations.

**Table 1:** Design table

Question	Hypothesis	Sampling plan (e.g. power analysis)	Analysis Plan	Interpretation given to different outcomes
Is the reaction time in the HLJT correlated with the muscle-specific increase in single-pulse MEPs during movement imagery?	The reaction time in the HLJT will be at least weakly and *negatively* correlated (r = −0.3) with the change in single-pulse MEP during ‘active’ movement imagery.	For a true Pearson’s r = −0.3, and a 95%CI width = −0.3 (r varies ± 0.15, from −0.15 to −0.45), the target sample size would be *n* = 140 individuals.	Sequential Bayesian Pearson’s correlation coefficients: BF_10_ > 10 and BF_01_ > 3 to obtain evidence in favour of H1 or H0, respectively.	If evidence is found for the presence of an association, it would imply biological validity for the use of the reaction time in the HLJT as a movement imagery ability test. If evidence is found for the absence of an association, it would imply lack of biological validity for the reaction time in the HLJT.
Is the reaction time in the HLJT correlated with the muscle-specific decrease in cortical inhibition (i.e. disinhibition) during movement imagery?	The reaction time in the HLJT will be at least weakly and *negatively* correlated (r = −0.3) with the decrease in cortical inhibition during ‘active’ movement imagery.	As above.	As above.	As above.
Is the kinaesthetic sum-score of the MIQ-RS correlated with the muscle-specific increase in MEPs during movement imagery, and muscle-specific decrease in cortical inhibition?	The kinaesthetic sum-score of the MIQ-RS will be at least weakly and *positively* correlated (r = −0.3) with the increase in single-pulse MEPs and the decrease in cortical inhibition, during ‘active’ movement imagery.	As above.	As above.	As above.
Is the difference in slopes of the CRFT correlated with the muscle-specific increase in MEPs during movement imagery, and muscle-specific decrease in cortical inhibition?	The difference in slopes will be at least weakly and *negatively* correlated (r = −0.3) with the increase in single-pulse MEPs and the decrease in cortical inhibition, during ‘active’ movement imagery.	As above.	As above.	As above.

Abbreviations: BF: Bayes Factor; CRFT: Chronometric Radial Fitts Task; HLJT: Hand Laterality Judgement Task; MIQ-RS: Movement Imagery Questionnaire-Revised Second Edition; MEPs: Motor Evoked Potentials.

## Materials and methods

### General procedures

A within-participants design will be used. The study will take place at the Institute of Neuroscience, UCLouvain (Belgium), and reporting will follow the Guidelines for Reporting Action Simulation Studies^40^ and checklists for TMS experiments.^41^ Ethical approval was obtained from the local Ethical Committee (Cliniques Universitaires Saint-Luc, Belgium; ID: NBBAS-2024/20DEC/566). All participants will provide written informed consent and receive financial compensation (€12.5/h).

### Participants

Healthy individuals aged 18–40 years, with normal or corrected-to-normal vision and no neurological or psychiatric history will be included (see power analyses below). Handedness will be determined via the Edinburgh Handedness Inventory,^42^ with a Laterality Quotient ≥40 indicating right-handedness (range: [−100, +100]).^43^ Eligibility for TMS will be screened with a standard self-administered questionnaire,^44^ excluding participants with epilepsy, metal implants, or other standard contraindications. Socio-demographic data will include age, gender, education, handedness, and prior experience with movement imagery, non-motor visual imagery, and reaching tasks.

### General procedure

PsychoPy software (version ≥2024.2.0) will present all stimuli.^45^ Stimulus size is expressed in ‘PsychoPy units’, where 1 unit equals screen height in landscape mode. Participants will complete a behaviooral battery of movement imagery tests and a neurophysiological TMS assessment, with order counterbalanced across participants. Before testing, a standardized sheet will explain movement imagery, visual versus kinaesthetic modalities, and first- versus third-person perspectives; the experimenter will clarify any doubts at that stage.

### Neurophysiological assessment of movement imagery

#### TMS general procedure

**Figure 1:**
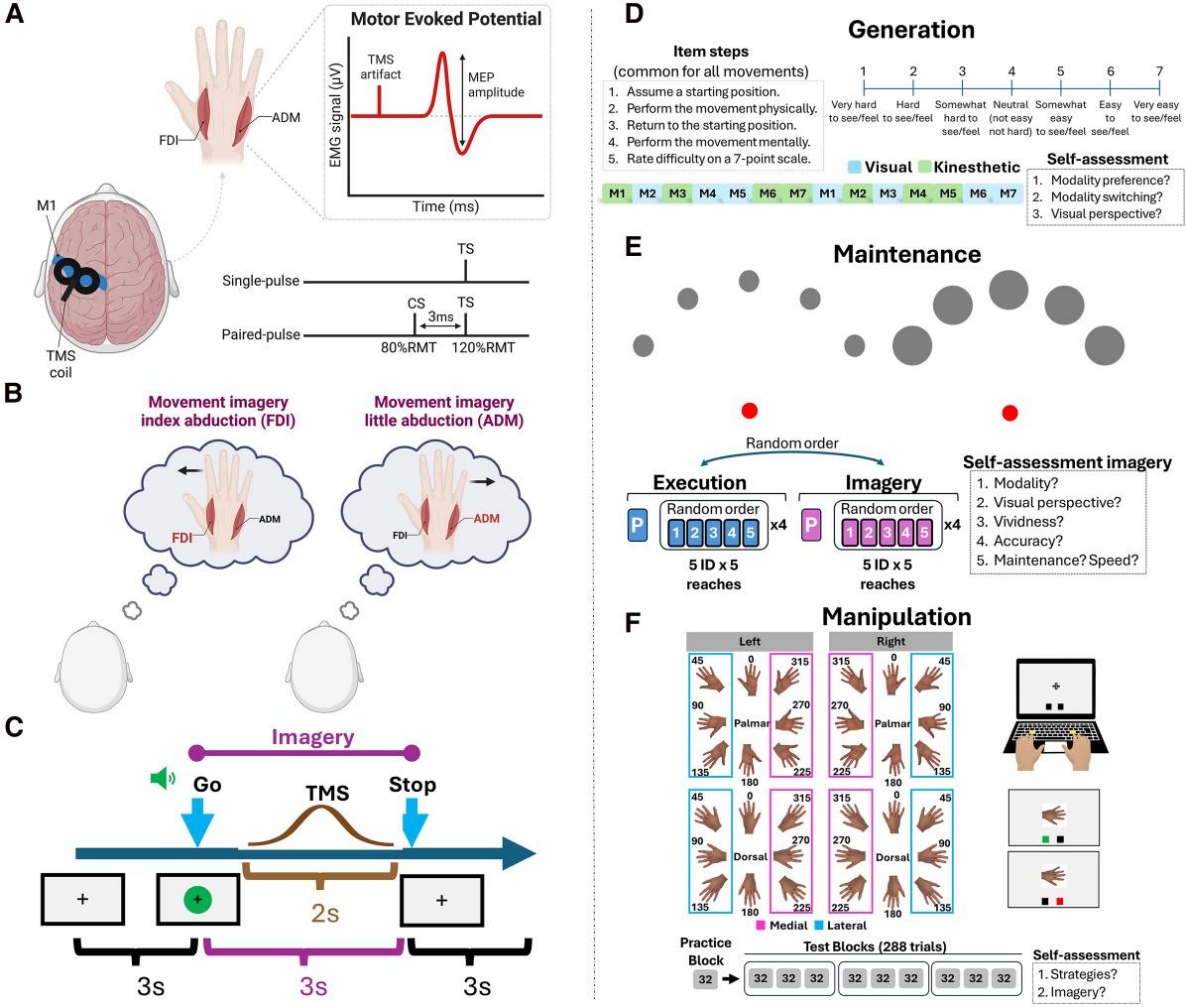
**Overview of the experimental methodology to assess movement imagery ability. Panels a–c** depict the neurophysiological assessment and **Panels d–f** the behavioural assessment. **Panel A** shows the general TMS procedure. Motor Evoked Potentials (MEPs) from the dominant First Dorsal Interosseous (FDI) and Abductor Digiti Minimi (ADM) will be recorded via Transcranial Magnetic Stimulation (TMS). Two TMS protocols (which will be randomly delivered) will be used. For single-pulse TMS, only the Test Stimulus (TS) is delivered, whereas in the paired-pulse TMS (SICI), the Conditioning Stimulus (CS) is delivered 3 ms before the TS, decreasing the MEP amplitude in response to the TS. Intensities for these stimuli will be set based on the participant’s Resting Motor Threshold (RMT). **Panel B** shows the two main experimental conditions (plus rest – not represented). During movement imagery, participants will imagine performing either an index finger abduction or a little finger abduction (maximal voluntary contraction of FDI and ADM, respectively, maintained for 3 s). TMS will be delivered during imagery in order to probe corticospinal excitability for both the ‘imagined muscle’ and the ‘non-imagined muscle’ in each trial. **Panel C** schematically illustrates trials in this experiment. A Go signal (arbitrary green circle accompanied by a sound) will appear to indicate to the participant that they should start the current imagery task. The stimulus will stay on screen for 3 s, indicating imagery must be maintained during this period. The TMS pulse can be delivered between 1–3 s after the Go signal, with a gaussian distribution to induce variability. **Panel D** shows the Movement Imagery Questionnaire-Revised Second edition to assess imagery generation. The questionnaire has 7 movements imagined in visual or kinaesthetic modalities. Participants will describe their modality preferences, unintentional use of visual/kinaesthetic modalities and visual perspectives after completing the questionnaire on 11-point rating scales. **Panel E** shows the Chronometric Radial Fitts’ Task to assess imagery maintenance. Participants will physically tap or imagine tapping (with a stylus) radially arranged circular targets with their dominant hand. Execution and imagery durations are isolated through simultaneous key presses with their non-dominant hand. Targets vary in difficulty according to Fitts’ law, which should hold for both execution and imagery. After the imagery block participants will describe their experience during imagery on 11-point rating scales. **Panel F** shows the Hand Laterality Judgement Task to assess imagery manipulation. Participants will see images of rotated hands in 8 possible angles and will be asked to judge their laterality (left or right), responding bimanually with their corresponding hand. Stimuli will be rotated clockwise or counterclockwise towards medial or lateral orientations. Feedback on accuracy will be provided throughout the task via two small boxes located at the bottom of the screen. Participants will describe their strategies and use of imagery on 11-point rating scales after finishing the task. Panels A and B were created in BioRender: [Robert Hardwick]. 2025. Link (Part A): https://app.biorender.com/biorender-templates/details/t-693fde2126c3ddda8ec5b095-tms-setup-meps/?source=gallery. Link (Part B): https://app.biorender.com/biorender-templates/details/t-693fe1033897dfd4bda6fe3c-movement-imagery-conditions/?source=gallery.

TMS will be used to elicit MEPs of the First Dorsal Interosseous (FDI) muscle and the Abductor Digiti Minimi (ADM) muscle of the dominant hand (Fig. 1A). We will record these two muscles to confirm muscle specificity of movement imagery and have an attention-matched condition. MEPs from these two muscles can be easily collected simultaneously with the same hotspot on a trial-by-trial basis.^46^ Additionally, as these two muscles do not have an agonist-antagonist relationship, comparisons between them will allow us to investigate effects of muscle specificity without confounds due to spinal mechanisms (i.e. reciprocal inhibition). The target location in the brain will be the ‘motor hotspot’, defined as the location of the Primary Motor Cortex (M1) that produces the largest and most consistent MEP amplitude in both FDI and ADM of the dominant hand using the lowest possible stimulation intensity. To find the optimal scalp position, the TMS coil will be positioned to induce a posterior-anterior current (coil handle facing backwards) and oriented approximately 45° rotated from the midline in the horizontal axis. MEPs will be determined as the peak-to-peak amplitude of the EMG signal (in μV) after the stimulus artefact. After determining the motor hotspot, the Resting Motor Threshold (RMT) will be calculated, defined as the minimum stimulation intensity at which MEPs of at least 50 μV amplitude in both FDI and ADM are produced in at least 5 out of 10 trials.^47^ RMT will be expressed as a percentage of the Maximum Stimulator Output.

The EMG signal of each muscle will be acquired with two circular Ag/AgCl self-adhesive surface electrodes (diameter = 9 mm) placed using a belly-tendon montage. A ground/reference electrode will be placed over the ulnar styloid process. The skin will be cleaned with alcohol before electrode placement. The EMG signal will be amplified with a gain of 1000, online bandpass filtered (1–1000 Hz) and Notch-filtered (50 Hz) by a Digitimer D360 amplifier (Digitimer Ltd, Welwyn Garden City, Hers, UK), and digitized at 4KHz by a Power 1401 unit (Cambridge Electronic Design Ltd., Cambridge, UK). EMG data will be acquired using Signal v6.04 (Cambridge Electronic Design Ltd., Cambridge, UK) and stored for offline processing and analysis (see Supplementary Materials for offline bandpass filtering and post-processing of EMG). Single-pulse and paired-pulse TMS protocols will be delivered using two Magstim 200^2^ monophasic stimulators coupled through a BiStim module and connected to a single figure-of-eight coil with 70-mm outer diameter (Magstim Co., UK). As 200^2^ and BiStim configurations are not equivalent in terms of maximum stimulator output,^48^ TMS will be applied always in BiStim mode throughout the experiment (including hotspotting, determining the RMT and single- and paired-pulse protocols), as it will be required for the paired-pulse protocol. Coil placement will be tracked throughout the experiment using a Visor2® Neuronavigation System version 2.5.3.50294 (ANT Neuro). This system allows us to track coil position in 3D space with accuracy of ∼0.1 mm. Accurate coil position will be ensured via online visual feedback of 3 parameters showing the deviation of the coil from the target hotspot (distance to target in mm, tilt deviation in degrees and rotation deviation in degrees). We will aim to keep all 3 parameters simultaneously below 3 units each throughout the experiment (i.e. <3 mm and <3°), which ensures precise coil positioning. Trial rejection criteria based on coil placement or signal noise are detailed in Supplementary Materials.

#### Experimental conditions

MEPs will be collected under three experimental conditions in single-pulse and paired-pulse TMS protocols (see below): (1) movement imagery of an ‘active’ muscle, (2) movement imagery of a ‘non-active’ muscle and (3) rest. Participants will be required to maintain their eyes open throughout the experiment. Stimuli will be presented on a 19-inch screen (refresh rate = 60 Hz) and controlled in PsychoPy, which will be combined with Signal to trigger TMS pulses. The participant will sit comfortably at approximately 60 cm from the screen, with their dominant hand resting on the desk.

***Movement imagery:*** The individual will be asked to imagine abducting their dominant index finger, therefore simulating a contraction of the FDI, or imagine abducting their dominant little finger, therefore simulating a contraction of the ADM. Because in every trial MEPs will be collected from both muscles (Fig. 1B), we anticipate that the imagined activation of a muscle will have specific effects for the corresponding muscle (i.e. MEPs of FDI should increase when imagining using the index but not the little finger, and MEPs of ADM should increase when imagining using the little, but not the index finger; see Supplementary Figs S1, 2 for pilot data). The individual will be instructed to imagine producing a ballistic movement with their maximal possible force (i.e. simulating a Maximal Voluntary Contraction) without actually producing any movement or perceptible contraction. Before imagery, they will be allowed to physically practice the finger abductions and will also be indicated that the movement must be of maximal amplitude (i.e. maximal Range of Motion available), enhancing the goal-directedness of the movement. Each trial, a fixation cross will be presented. The individual will be cued with an arbitrary on-screen Go signal (a green circle of 0.2 × 0.2 PsychoPy units placed at the centre of the screen–see Fig. 1C) accompanied by a short beep (200 ms). The individual will be asked to simulate a ballistic movement of the finger as soon as the Go signal appears and maintain it until the circle disappears (i.e. a concentric phase followed by an isometric phase). Previous evidence suggests that kinaesthetic and visual modalities of movement imagery may have different neural substrates, the former showing stronger activation of classical motor-related areas.^49,50^ It has also been suggested that kinaesthetic imagery leads to stronger increases in corticospinal excitability than visual imagery.^51^ Therefore, the participant will be instructed to focus on kinaesthetic aspects of imagery, with the instruction ‘imagine the feeling of the action, focusing on sensations like the contraction of the muscle and the movement of the joint’. Instructions will be given on-screen and standardized across participants. Even if indicated to use kinaesthetic imagery only, participants might experience difficulties to isolate a single sensory modality. Therefore, post-experiment self-assessments will be collected to assess the use and vividness of the different modalities and perspective of imagery (see below and Supplementary Materials).

***Rest:*** Participants will be asked to maintain a relaxed position without any explicit cognitive task, while keep looking at the fixation cross. The same arbitrary cue will be employed as in the imagery condition.

#### TMS protocols (Fig. 1A)

For single-pulse TMS, a single Test Stimulus (TS) will be delivered over M1, to obtain MEPs in the two target muscles in each experimental condition. For the TS, stimulation intensity will be set at 120%RMT to obtain reliable MEPs throughout the experiment.^52^ The paired-pulse TMS will be a Short Interval Cortical Inhibition (SICI) protocol.^53,54^ In this protocol, the TS will be preceded by a Conditioning Stimulus (CS) delivered 3 ms before the TS, through the same coil. For the CS, a subthreshold intensity of 80%RMT will be employed.^55^ In both protocols, the first pulse (TS or CS) will be delivered at a random time between 1–3 s after the on-screen Go signal. The timings for each participant will have a Gaussian distribution with mean = 2 s and SD = 0.25 s, which will be the same for each TMS protocol and experimental condition, but whose order will be randomized independently for each condition. This distribution has been chosen to induce variability in the exact timing of the TMS pulse across trials, to avoid participants predicting the moment at which the pulse would occur.

#### Randomization and counterbalancing

A total of 30 trials will be collected for each condition – movement imagery of the FDI muscle, movement imagery of the ADM muscle, and rest – and for each TMS protocol (single-pulse and SICI-conditioned), ensuring reliable MEP amplitude measures.^56^ The inter-trial interval will be at least 3 s to allow stimulator recharge, prevent coil overheating, and avoid trial-to-trial carry-over effects.^57^

Data will be collected in blocks of 60 trials, evenly divided across conditions. Within each block, participants will complete sub-blocks of 10 consecutive trials of the same condition. Sub-block changes will be signalled by on-screen text and a short tone. Condition order within each block will be pseudo-randomized so that every three sub-blocks include one of each condition and no condition is repeated consecutively. This design controls for time-related fluctuations in corticospinal excitability while minimizing fatigue or confusion.

Each sub-block of 10 trials will contain 5 single-pulse and 5 paired-pulse (SICI) trials, presented in random order with no more than 3 consecutive trials using the same TMS protocol. Participants will complete 180 trials in total (30 trials × 3 conditions × 2 protocols). To reduce fatigue, a rest period of at least 1 min will be provided between blocks.

#### Attention checks

Because there are no quantitative performance indicators we can measure during imagery, we will use attention checks at the end of randomly selected sub-blocks where participants must indicate which task they are performing, to ensure attention is maintained. Each grand block, 2 attention checks will be collected (i.e. 6 overall). Note that every trial the participant will be reminded of the current task they need to perform via on-screen text (imagery of FDI, imagery of ADM, or rest), making it very unlikely to miss these attention checks.

#### Self-report imagery vividness

With the aim of determining possible direct relationships between corticospinal excitability and traditional self-report measures of imagery ability, at the end of each block participants will be asked to rate their kinaesthetic imagery vividness during movement imagery trials. They will do it on 11-point scales (0 = ‘No image at all, I only know I am thinking about the task’ to 10 = ‘Very intense as normal feeling of movement’).

#### Qualitative reports (self-assessments)

As this will be the largest TMS study collecting data during movement imagery so far, we will ask participants qualitative questions for purely descriptive analyses which could inform the design and interpretation of future studies. The questions will focus on aspects such as the use of visual or kinaesthetic modalities of imagery, their vividness, the content of imagery, muscle specificity during imagery, etc. These questions are listed in Supplementary Materials and data derived from them will only be presented descriptively, without any formal statistical analysis.

### Behavioural assessment of ‘movement imagery ability’

#### Generation

The ability to generate movement imagery will be assessed using the Movement Imagery Questionnaire–Revised Second Edition (MIQ-RS).^58^ This questionnaire is developed for both healthy and clinical populations, enabling replication in individuals with motor impairments. It has also been translated and cross-culturally adapted into multiple languages,^59,60^ ensuring generalisability.

The MIQ-RS (Fig. 1D) is a 14-item self-administered tool assessing 7 movements in two sensory modalities: visual and kinaesthetic. Each item involves: (1) adopting an initial position; (2) physically performing a movement; (3) returning to the initial position; and (4) visually or kinaesthetically imagining the movement. Participants rate the ease or difficulty of generating the image on a 7-point Likert scale (1 = very hard to see/feel; 7 = very easy to see/feel). Visual and kinaesthetic items are interspersed. Scores can be reported as a total (14–98 points) or by subscale sum-scores (visual or kinaesthetic, each 7–49 points), with higher scores indicating better imagery ability.

Originally developed in English, the MIQ-RS shows good psychometric properties, including robust factor structure and test–retest reliability in healthy and clinical groups. As the study will be conducted in a French-speaking community, we will use the French version for most participants,^59^ while fluent non-francophones will complete the original English version.^58^ An attention check will be embedded mid-questionnaire to ensure proper completion. Afterward, participants will provide self-assessments on imagery preferences (visual versus kinaesthetic), perspective (first- versus third-person), and related experiences (see Supplementary Materials).

#### Maintenance

The ability to create a temporally accurate action representation will be assessed using the Chronometric Radial Fitts Task (CRFT), a novel method based on Fitts’ law,^61^ which links movement difficulty and duration. It measures how well this relationship is preserved in movement imagery. Participants use a stylus to physically tap, and imagine tapping, radially arranged circular targets with their dominant hand (Fig. 1E). Execution and imagery durations are recorded via simultaneous key presses with the non-dominant hand. Target difficulty varies per Fitts’ law, which should hold for both execution and imagery in individuals with good imagery maintenance (see Supplementary Fig. 3 for pilot data).

The task will be performed on a 24-inch capacitive touchscreen using a capacitive stylus.^62^ Five indices of difficulty (ID) will be defined by the diameter of five grey targets (0.018, 0.024, 0.05, 0.1, 0.21 PsychoPy units), corresponding to IDs of 6.34, 5.35, 4.3, 3.38, and 2.47. All targets are radially arranged at a constant edge-to-edge distance (0.4 units) from a fixed red ‘home’ target (diameter 0.05, location (0, −0.2) in PsychoPy units). Using edge-to-edge distance ensures that reach time from home to each target (5 reaches/trial) increases linearly with ID.

In each trial, participants alternately tap the home target and each grey target, starting and ending at home (11 taps total), moving from their non-dominant to dominant side (e.g. left-to-right for right-handed). Simultaneously, they press the space bar with the non-dominant index finger to record duration in both execution and imagery (no physical taps in imagery).

Participants will complete two conditions (execution, imagery), each with 4 repetitions per ID (4 × 5 IDs = 20 trials × 5 reaches = 100 measurements per condition). Conditions will be blocked (two blocks of 20 trials) and block order randomized across participants. IDs will be randomized within blocks. Before each block, a 5-trial practice with moderate IDs will provide feedback on total duration to familiarize participants. In imagery trials, participants will use both visual and kinaesthetic modalities, keeping eyes open. After the imagery block, they will self-assess their experience (see Supplementary Materials). A minimum 1-minute rest will be provided between blocks.

#### Manipulation

The ability to transform the content of a mental action representation will be assessed with the HLJT, using a recent open-source paradigm.^63^ Participants decide whether a stimulus shows a left or right hand (Fig. 1F). Left-hand images are mirror-reversed right-hand images. Stimuli appear in 8 frontal rotational angles (0°, 45°, 90°, 135°, 180°, 225°, 270°, 315°; clockwise for right hands, counterclockwise for left hands) and 2 views (palmar or dorsal), totalling 32 unique stimuli. The biomechanical constraints effect, indicative of motor processing,^64,65^ measures how biological limitations influence imagery in this task: medial (towards midline) rotations are processed faster than lateral (away from midline) rotations, calculated as the reaction time difference between medial and lateral rotations.

Each trial begins with an 800 ms central fixation cross. Stimuli (0.45 × 0.45 PsychoPy units) are presented until a response is made. Participants respond bimanually, with left/right index fingers on the ‘S’/‘L’ keys. Visual feedback is shown for 300 ms via two small boxes (0.07 × 0.07 units) at the screen bottom, turning green for correct and red for incorrect responses.

A practice block with 32 trials (1 repetition per unique stimulus) familiarizes participants, followed by 3 test blocks of 96 trials each (3 repetitions per stimulus). Within each test block, stimuli are randomized in sub-blocks of 32 trials to avoid repeating the same stimulus more than twice consecutively. Only test blocks are analysed, giving a total of 288 trials per participant (9 repetitions per stimulus). Participants can rest for at least 1 min between blocks.

#### Qualitative reports (self-assessments)

As this will be the largest behavioural study collecting data during movement imagery alongside TMS-derived data to date, we will ask participants qualitative questions for purely descriptive analyses which could inform the design and interpretation of future studies. The questions will focus on aspects such as the use of visual or kinaesthetic modalities of imagery, their vividness, the content of imagery, their ability to generate, maintain and manipulate it, its speed, etc. These questions are listed in Supplementary Materials and data derived from them will only be presented descriptively, without any formal statistical analysis. They are specific for each behavioural test of the battery.

### Individual-level outcome measures

Defining appropriate individual-level outcome measures is necessary to then use them for subsequent correlation analyses. Below we describe these measures in detail.

#### Neurophysiological measures

***Corticospinal excitability:*** taking data from the single-pulse TMS protocol only, we will obtain the MEP amplitude in the ‘active’ movement imagery condition (presumed to reflect participants’ imagery ability) and normalize it. As absolute MEP amplitudes may have significant inter-individual and inter-muscle variability, the choice of the most appropriate baseline condition to normalize by is an important aspect of TMS experiments.^66,67^ In our case, we could consider the rest condition or the ‘non-active’ movement imagery condition. There is compelling evidence that movement imagery produces muscle-specific changes in corticospinal excitability^26,27,35,68-70^ – for an overview see.^71^ Therefore, although for group-level analyses the rest condition would be the most sensible baseline (as it allows to directly statistically compare the increase in corticospinal excitability between ‘active’ and ‘non-active’ imagery conditions), at the individual level this would ignore the fact that corticospinal excitability could be increased in a muscle-unspecific manner. That scenario would not necessarily reflect ‘better’ movement imagery ability, as the increase in MEP would not be specific to the muscle being imagined. Therefore, normalizing by the ‘non-active’ imagery condition provides a direct measure of muscle-specific increases in corticospinal excitability, which is a straightforward metric of movement imagery ability. We note that normalizing by the ‘non-active’ imagery condition has the potential limitation of ignoring the fact that both ‘active’ and ‘non-active’ conditions could show smaller MEPs in comparison to the rest condition (i.e. illustrating an inhibitory effect of imagery overall – though we note this effect is not present in our pilot data at the overall group level). However, we will normalize the data by the ‘non-active’ imagery condition as it is conceptually clearer than normalizing by rest, accounts for general (non-muscle-specific) increases in corticospinal excitability during imagery, and a large body of evidence suggests the effect of imagery should be muscle-specific. We will use the formula: % MEP change = (MEP_active_/MEP_non-active_) × 100. In this measure, muscle-specific corticospinal facilitation during movement imagery will be illustrated by %MEP changes > 100%, larger values indicating greater facilitation. A result of 100% will mean no corticospinal facilitation effect (i.e. poor imagery ability as the effect is not muscle-specific).

***Intracortical inhibition:*** in SICI paradigms (and other paired-pulse TMS protocols), the most widely implemented metric compares the average amplitude of conditioned MEPs (paired-pulse TMS protocol) with the average amplitude of unconditioned MEPs (single-pulse TMS protocol),^72^ using the formula: % Inhibition (%INH) = (MEP_conditioned_/MEP_unconditioned_)*100. There is also evidence that movement imagery leads to muscle-specific inhibition.^73^ Therefore, for consistency with our previous approach and with available evidence, we will obtain the individual-level outcome measure by comparing the %INH of the ‘active’ imagery condition with the ‘non-active’ imagery condition. As both measures are already in the % metric, a simple subtraction (%INH_active_ – %INH_non-active_) is straightforward to interpret, as positive values indicate muscle-specific cortical disinhibition, and values close to 0 or negative indicate general cortical disinhibition, which illustrate good and poor movement imagery ability, respectively.

#### Behavioural measures

***MIQ-RS*:** As previous studies have reported significant correlations between corticospinal excitability and imagery questionnaires mainly for the kinaesthetic modality, and participants will perform kinaesthetic imagery in the TMS experiment, only the kinaesthetic sum-score will be used for confirmatory analysis. Being a bounded metric, it will be normalized to a 0–100% scale: Kinaesthetic score = ((Sum-score – Minimum)/Range) × 100. Higher values indicate better imagery ability.

***CRFT*:** This task evaluates how well Fitts’ law is preserved in movement imagery compared to execution, reflecting the ability to sustain imagery over time. Movement time should increase linearly with ID; thus, for each condition (execution, imagery), a simple linear regression (Reach Time ∼ ID × Condition) will yield slopes. A Gamma link will be used to account for right-skewed times. The difference between back-transformed slopes (in ms) between execution and imagery will be taken as the absolute value, with values closer to 0 indicating better imagery ability. As a secondary measure, we will also consider the y-intercept, representing movement duration for an Index of Difficulty = 0. We note the y-intercept would be primarily informative if the execution and imagery slopes are not parallel, and it expected to be correlated with the slope. As index of imagery ability, we will compute the difference between y-intercepts between execution and imagery, with values closer to 0 indicating better imagery ability.

***HLJT*:** Although both reaction time and accuracy can be measured, accuracy typically shows a ceiling effect (∼90% correct).^63^ Therefore, the confirmatory outcome will be overall reaction time (ms) from correct trials, averaged across all conditions (rotation angles, hand views, directions). Lower reaction times indicate better imagery ability.

### Sample size calculations

The study’s primary contrast is the correlation between corticospinal excitability (change in MEP amplitude during single-pulse TMS in the movement imagery condition) and overall reaction time in the HLJT. This correlation was selected because prior studies have reported it,^39^ enabling direct comparison, and because HLJT is a more precise and objective measure than other behavioural tests.

Previous work found r = −0.56 for HLJT reaction time versus MEP change (r = 0.31 for accuracy), and r = 0.65 for the kinaesthetic subscale of VMIQ-2.^39^ Other studies reported no correlation with total MIQ-R scores (r not given) but found r = 0.47 with trial-to-trial vividness,^37^ or correlations with kinaesthetic subscales depending on the questionnaire (KVIQ: r = 0.61; VMIQ-2: r = 0.36).^38^ A ‘motor imagery index’ combining questionnaires, mental chronometry, and physiological data showed a weak correlation with MEPs (r = 0.23).^27^ A meta-analysis concluded that the MEP effect of combined action observation and movement imagery was mainly due to imagery, and kinaesthetic scores did not moderate it (β = −0.01).^74^

Given this mixed evidence, we will sample to detect small correlations (r = 0.3 in either direction).^75^ This is conservative, as stronger correlations in prior work came from small samples (*n* < 25). In a frequentist NHST framework (two-tailed, α = 0.05, power = 0.95), *N* = 139 participants would be required (pwrss v0.3.1 in R v4.4.2).^76^ Precision-based calculations for r = 0.3 and 95% CI width = 0.3 also yielded *n* = 140 (MBESS v4.9.3; presize v0.3.7).^77^ Thus, both approaches converge on *N* = 140 as adequate.

We will use a Bayesian framework to obtain evidence for the null hypothesis (H0) or the alternative hypothesis (H1). Data will be collected until Bayes Factor (BF) ≥ 10 for H1 (‘Strong’ evidence) or BF ≥ 3 for H0 (‘Moderate’ evidence). Thresholds are asymmetric because BF ≥ 10 for H0 would require ∼2000 participants, which is unfeasible for the planned study. According to our simulation-based Bayesian Power Analysis (see Supplementary Materials), with r = 0.3, *N* = 140 yields BF > 10 for H1 with 77% probability and BF > 3 for H0 with 68% probability (Supplementary Fig. 4). Calculated with BayesFactor v0.9.12-4.7^78^ and correlation v0.8.6^79^ packages.

As evidence may emerge before reaching *N* = 140, a sequential stopping rule^80,81^ will be applied: a minimum of 70 participants will be tested, then data will be reviewed every 10 participants until: (A) BF ≥ 10 for H1, (B) BF ≥ 3 for H0, or (C) *N* = 140 is reached. Multiplicity in Bayesian sequential analyses will be controlled via progressive prior shrinkage (see Supplementary Materials and Supplementary Fig. 5 for details).^82^

### Statistical analysis

Analyses will be conducted in R v4.4.2 or superior (R Core Team 2026). Continuous variables will be summarized as mean ± SD, categorical as *n* (%). Supplementary Materials detail data pre-processing, manipulation checks, and methodological checks.

#### Primary confirmatory analysis

Given multiple possible outcome measures, selecting a primary behavioural–neurophysiological correlation is challenging. The HLJT, widely used as an implicit, objective measure of movement imagery (reaction time, accuracy), has been chosen over the MIQ-RS (subjective self-report) and CRFT (semi-objective, novel, with limited neurophysiological validation). Both MIQ-RS and CRFT will be analysed as secondary confirmatory hypotheses. The main correlation will be between %MEP change (‘active’ versus ‘non-active’ imagery) in single-pulse TMS and HLJT reaction time, using Bayesian Pearson’s correlation (‘correlation’ v0.8.6).^79^ Relationships will be visualized with scatter plots, posterior distributions, and interpreted as negligible (<0.1), weak (0.1–0.4), moderate (0.4–0.7), strong (0.7–0.9), or very strong (>0.9).^83^ Spearman’s correlation will be used in sensitivity analyses to relax normality assumptions. Parameter uncertainty will be expressed as 95% Credible Intervals (95%CrI).

For each correlation, three Bayesian indices will be reported:^84^

**Bayes Factors (BFs):** Calculated via the Savage–Dickey density ratio,^85^ presented as BF_01_ (for H0) or BF_10_ (for H1), and interpreted as inconclusive evidence (=1), anecdotal (1–3), moderate (3–10), strong (10–30), very strong (30–100), or extreme (>100) evidence.^86,87^ The first ‘data look’ (*N* = 70) will use a non-informative Cauchy prior for the correlation coefficient (centre = 0, rscale = 1). Priors will be progressively shrunk at each look to control for multiplicity.^78,88^

**Probability of Direction:** Proportion of the posterior off the median’s sign, interpreted as the probability the parameter is strictly positive or negative (range: 50–100%).^84^

**Region of Practical Equivalence (ROPE) Percentage:** Proportion of the 95% Highest Density Interval within the ROPE (−0.1 to 0.1 for r), indicating trivial/negligible correlations.^89^

#### Secondary confirmatory analyses

An equivalent procedure as described above will be followed for the rest of the comparisons. We will correlate the corticospinal facilitation measure with the kinaesthetic subscale of the MIQ-RS, the difference in slopes and y-intercept measures of the CRFT and the direct vividness ratings provided during the TMS experiment. Finally, the intracortical disinhibition measure will be correlated with the three behavioural measures of movement imagery ability and the direct vividness ratings.

### Pilot data

We provide pilot data showing the feasibility of our TMS experiment and the novel CRFT task. The details are reported fully in Supplementary Materials.

In brief, for the TMS experiment we collected data from 10 healthy individuals (5 females, 5 males; 9 right-handed, 1 left-handed; age = 26.7 ± 2.53 years (mean ± SD), range = 22–30 years; RMT = 53.5 ± 7.46% MSO, range = 38–62% MSO). In single-pulse TMS (Supplementary Fig. 1B), the imagined (‘active’) muscle showed an average increase in z-scored MEP amplitude compared to rest with a moderate-to-large effect size (Cohen’s d (*d_rm_*) = 1.05, 95% confidence interval [−0.32, 2.42]) and compared to the not imagined (‘non-active’) muscle (*d_rm_* = 0.71 [0.29, 1.13]). The non-active muscle showed a weaker facilitatory effect compared to rest (*d_rm_* = 0.34 [−0.8, 1.49]). Additionally, compared to unconditioned MEPs (single-pulse TMS), conditioned MEPs (paired-pulse TMS) showed smaller MEPs across all conditions (Supplementary Fig. 2), validating our SICI protocol. Compared to rest, the ‘active’ imagery condition showed less inhibition, with a moderate effect size and large uncertainty (*d_rm_* = 0.6 [−0.1, 1.31]). However, compared to the not imagined muscle (‘non-active’ imagery condition), the effect was negligible (*d_rm_* = 0.11 [−0.19, 0.40]), indicating that disinhibition during movement imagery may occur through a general (not muscle-specific) mechanism. Again, this proves feasibility of our proposed approach.

For the CRFT, we tested 10 healthy individuals (6 females, 4 males; 9 right-handed, 1 left-handed; age = 26.44 ± 3.03 years, range = 22–30 years; 8 participants overlapping with our pilot data from the neurophysiological assessment). The data replicate the fundamental effect whereby in the execution condition (Supplementary Fig. 3A), the group-level slope is different than 0 (Slope = 43.9 ms [29.6, 58.3]) and individual-level slopes vary from 31 to 79 ms (Supplementary Fig. 3B), showing a consistent increase of reaching time with difficulty. For imagery (Supplementary Fig. 3A), the group-level slope is also different than 0, although with a wider confidence interval (Slope = 38 ms [20.12, 55.9]), and individual-level slopes vary from 11 to 98 ms, illustrating different degrees of movement imagery ability in the sample.

Overall, our pilot data proves feasibility that the novel paradigms can be implemented in our laboratory.

## Supplementary Material

fcag013_Supplementary_Data

## Data Availability

All experiment materials, the raw and processed data, the code used for analysis, the data usage guidance, and the laboratory log documenting the details of data collection will be available via the Open Science Framework at https://osf.io/yujvt/. No data for any preregistered study (other than pilot data included at Stage 1) will be collected prior to the date of acceptance in principle. All data files will be collected after acceptance in principle and appropriately time-stamped according to the approved registered Stage 1 protocol.
