# Supplementary material for: The relationship between corticospinal excitability and behavioural measures of movement imagery ability
Source: Brain Commun. 2026 Jan 16;8(1):fcag013. doi: 10.1093/braincomms/fcag013 (PMC12895273; doi:10.1093/braincomms/fcag013)
Supplement: fcag013_Supplementary_Data [file fcag013_supplementary_data.pdf]

Supplementary Materials

Pilot data

Because imagery is a covert cognitive process, our study requires that both neurophysiological and behavioural assessments show evidence of adequate internal validity (i.e., fundamental effects of our experimental manipulations should be detectable at the group level). This is a 'methodological check' before considering correlations between them, an analysis conducted at the individual level, as pairs of data are related within each individual. Below, we provide justifications based on pilot data or well-established effects that demonstrate the feasibility of our proposed approach.

Neurophysiological measures

Our experimental protocol is unique, though heavily informed by previous studies.[1–7] We provide pilot data (N=10) replicating the effects of single-pulse TMS and paired-pulse TMS on MEPs during movement imagery (following identical procedures as outlined in the methods section and in Figure 1a-c). We have tested 10 healthy individuals (5 females, 5 males; 9 right-handed, 1 left-handed; age = 26.7 ± 2.53 years (mean ± SD), range = 22 – 30 years; Resting Motor Threshold (RMT) = 53.5 ± 7.46% MSO, range = 38 – 62% MSO).

<u>Corticospinal facilitation (single-pulse TMS):</u> First, we replicate the well-established phenomenon of corticospinal facilitation during movement imagery, as shown by an average increase in MEP amplitude in response to single-pulse TMS. Data were collected following the exact procedure as indicated above. In single-pulse TMS (Supplementary Figure 1), the imagined ('active') muscle showed an average increase in z-scored MEP amplitude compared to rest with a moderate-to-large effect size (Cohen's d (d_{rm}) = 1.05, 95% confidence interval [-0.32, 2.42]) and compared to the not imagined ('non-active') muscle (d_{rm} = 0.71 [0.29, 1.13]). The non-active muscle showed a weaker facilitatory effect compared to rest (d_{rm} = 0.34 [-0.8, 1.49]). We note the uncertainty intervals are large, as expected at this small sample size. However, the global trend provides proof-of-concept that the main effect of movement imagery on muscle-specific corticospinal excitability can be reproduced in our laboratory, even if the effect size might be overestimated at this sample size. This is essential for subsequent correlation analyses. Furthermore, pilot data suggest varying degrees of corticospinal facilitation during movement imagery at the individual level (Supplementary Figure 1C), which may be associated with individuals exhibiting varying levels of movement imagery ability (not measured behaviourally in the pilot sample).

<u>Cortical inhibition (paired-pulse TMS):</u> Second, we have also collected paired-pulse TMS data to assess intracortical inhibition (SICI)—see Fig. 1A and the procedure described above for detailed information. We note this effect has been less replicated in the literature, but a specific prediction is that movement imagery will reduce inhibition compared to rest,[1–5] though this effect has been varied across previous studies.[8–11] In our pilot data, compared to unconditioned MEPs (single-pulse TMS), conditioned MEPs (paired-pulse TMS) showed smaller MEPs across all conditions (Supplementary Figure 2), validating our SICI protocol. Compared to rest, the 'active' imagery condition showed less inhibition, with a moderate effect size and large uncertainty (d_{rm} = 0.6 [-0.1, 1.31]). However, compared to the not imagined muscle ('non-active' imagery condition), the effect was negligible (d_{rm} = 0.11 [-0.19, 0.40]), indicating that disinhibition during movement imagery may occur through a general (not muscle-specific) mechanism. Again, this proves feasibility of our proposed approach.

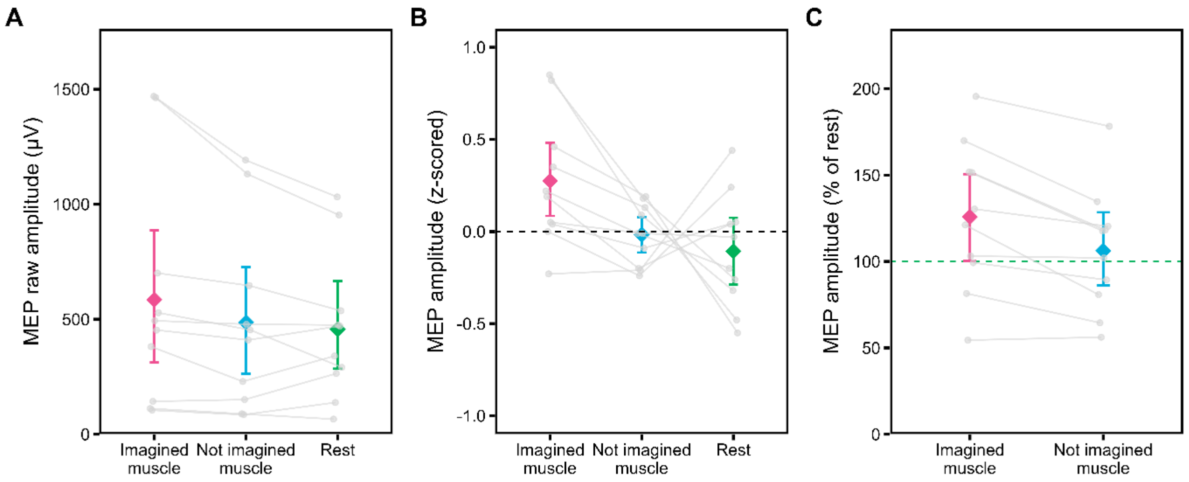

Supplementary Figure 1. *Pilot data (N=10). In all three panels, the peak-to-peak amplitude of Motor Evoked Potentials (MEPs) collected using single-pulse Transcranial Magnetic Stimulation (TMS) are shown in either their raw form, or following different normalization procedures, to help demonstrate the differences between these possible approaches for data analysis. MEPs are collected from the First Dorsal Interosseous (FDI) and the Abductor Digiti Minimi (ADM) muscles of the dominant hand. The contraction of each muscle was simulated by the participants using kinesthetic movement imagery. As MEPs from both muscles were measured during movement imagery in every trial, but only one muscle was imagined to be active at a time, we show results as the imagined muscle ('active' imagery condition) muscle as well as the 'not imagined muscle' (an attention matched but 'non-active' imagery condition), regardless of which specific muscle this was. Additional data was collected at rest, with no cognitive or physical task. Note that the plots are not faceted by muscle as the contribution of each specific muscle is not relevant for the purpose of the study—only whether they correspond to the imagined ('active') or not imagined ('non-active') in each imagery condition.* ***A*** *shows the raw MEPs for each imagery condition and at rest. Please note that raw MEPs are subject to inherent large inter-individual and inter-muscle variability, which may create artifacts or show spurious relationships. Because of this variability, previous TMS studies have proposed a number of different data processing procedures. B shows a transformation whereby the data for each participant has been normalized using z-scores. Z-scored data are useful for group-level comparisons as they eliminate inter-individual and inter-muscle variability, but can be difficult to interpret at the individual-level. It is also common practice in TMS experiments to report normalised MEPs as 'change from a baseline condition', which is a more easily interpretable metric. Because of that,* ***C*** *shows normalised MEPs for each imagery condition compared to rest (dashed horizontal blue line shows mean average MEP amplitude at rest for each participant), using the formula: (Imagery/Rest)*100. We note that for each participant, MEPs from each muscle are normalised by their own rest to account for between-muscle variations and then averaged. In this outcome measure, values greater than 100% indicate facilitation and lower than 100%, inhibition. In all panels, error bars show 95% confidence intervals using 1,000 bootstrapped samples, diamonds show average across participants for each condition, and grey dots and lines show individual participants.*

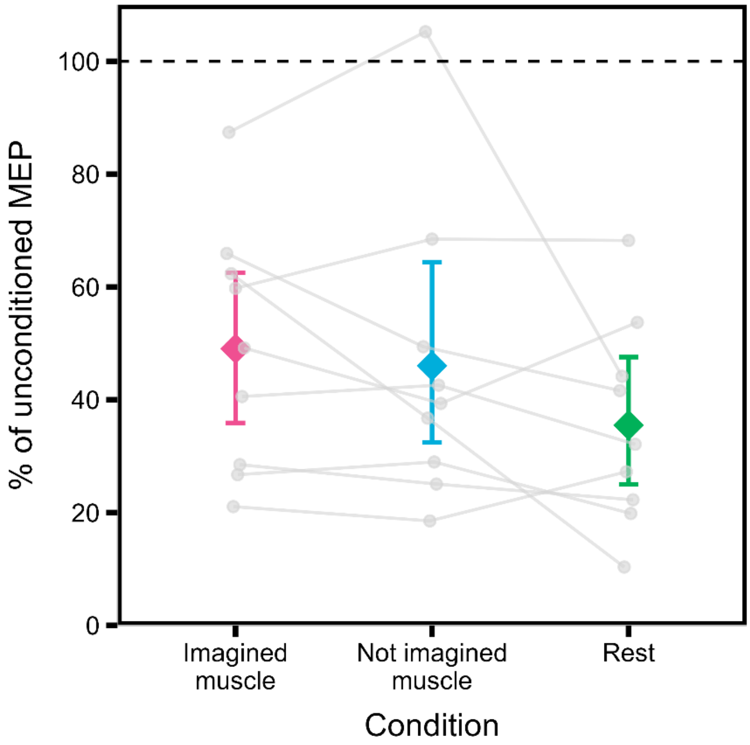

Supplementary Figure 2. *Pilot data (N=9) showing the effect of movement imagery on Short Interval Intracortical Inhibition (SICI). The outcome variable is the percentage ratio from the unconditioned Motor Evoked Potential (MEP), which compares the paired-pulse (conditioned) MEP with the single-pulse (unconditioned) MEP, using the formula: % Inhibition (% INH) = (Conditioned MEP/Unconditioned MEP)*100. In this measure values lower than 100% indicate inhibition and higher than 100% indicate facilitation, with 100% indicating no effect of the conditioning pulse. This makes inhibition comparable across participants and across imagery and rest conditions. For each participant, the data for each condition is the average of the two muscles from which MEPs were collected (FDI and ADM of the dominant hand), as the specific contribution of each muscle is not relevant for the purpose of the analysis, only if they correspond to the 'active' or 'non-active' imagery conditions. Data is shown from N=9 participants, as one participant had to be excluded due to a technical issue. Error bars show 95% confidence intervals using 1,000 bootstrapped samples, diamonds show average across participants for each condition, and grey dots and lines show individual participants.*

Behavioural measures

We have developed a battery of behavioural measures in order to provide a comprehensive assessment of imagery generation, maintenance and manipulation.

Imagery generation (Movement Imagery Questionnaire-Revised Second edition—MIQ-RS): While there are multiple questionnaires that assess movement imagery ability, the MIQ-RS (Fig. 1d) is chosen because there is ample evidence that its factor structure can distinguish between visual and kinesthetic sensory modalities of movement imagery,[12] and because of its relevance in both healthy and clinical populations. As there are no specific manipulation checks to be carried out, we do not provide specific pilot data in this regard.

Imagery maintenance (Chronometric Radial Fitts Task—CRFT): The CRFT is a novel approach based on the well-established relationship between a movement's difficulty and its duration, as stated in Fitts' law.[13] Our protocol will follow the same procedure as described in Czilczer et al. 2025.[14] However, because this task is relatively new, we provide pilot data. We tested 10 healthy individuals (6 females, 4 males; 9 right-handed, 1 left-handed; age = 26.44 ± 3.03 years, range = 22 – 30 years; 8 participants overlapping with our pilot data from the neurophysiological assessment). Our data replicate the fundamental effect whereby the time to physically tap or imagine tapping to the targets increases linearly with the Index of Difficulty (derived by modifying target width). In the execution condition (Supplementary Figure 3A), the group-level slope is different than 0 (Slope = 43.9ms [29.6, 58.3]) and individual-level slopes vary from 31 to 79ms (Supplementary Figure 3B), showing a consistent increase of reaching time with difficulty. For imagery, the group-level slope is also different than 0, although with a wider confidence interval (Slope = 38ms [20.12, 55.9]), and individual-level slopes vary from 11 to 98ms, illustrating different degrees of movement imagery ability in the sample. This provides feasibility that the paradigm can be implemented in our laboratory.

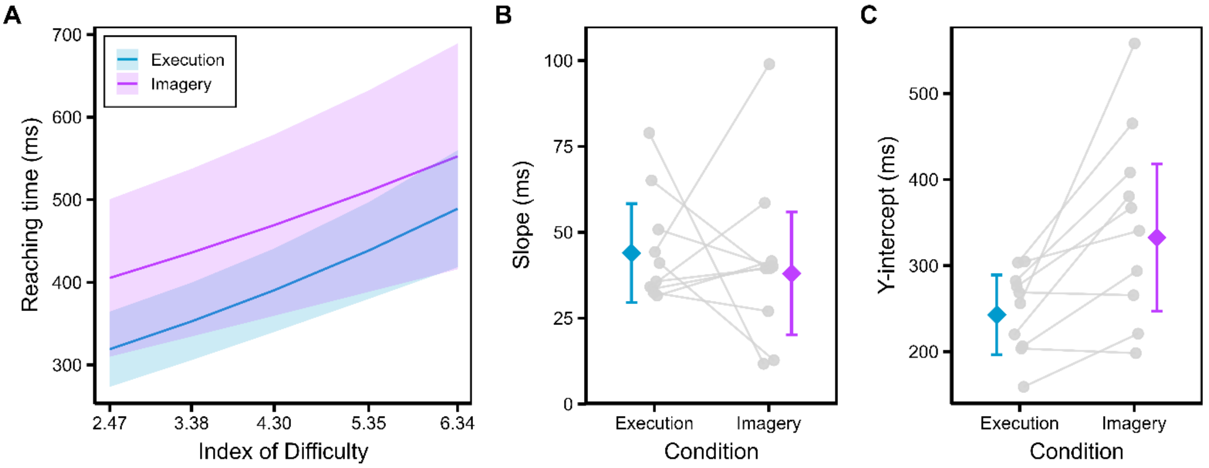

Supplementary Figure 3. *Pilot data for the Chronometric Radial Fitts' Task (CRFT).* ***Panel A*** *shows the group-level predictions for reaching time across the levels of difficulty, in execution and imagery conditions. It shows increases of reaching time as a function of Index of Difficulty (ID) in both conditions. However, the imagery condition shows a much wider confidence interval, which indicates differing movement imagery abilities in the sample.* ***Panels B and C*** *show the group-level slopes and y-intercepts with their 95% confidence intervals; grey points show individual-level data. Both panels show model-predicted values from a generalised mixed model with formula: Reach Time ~ ID * Condition + (1 + ID*Condition | Participant). At the individual-level, the difference of the execution and imagery slopes and y-intercepts is the outcome measure. The absolute difference (directionless) is used, values closer to 0 indicating better imagery ability.*

<u>Mental rotation task for manipulation (Hand Laterality Judgement Task—HLJT):</u> Our protocol will follow the same procedure as described in Moreno-Verdú et al. 2025.[15] This study provided an open-source HLJT protocol (8 angles of rotation in increments of 45°, with palmar and dorsal hand stimuli). The study showed evidence for validity and consistency of fundamental effects (stimulus rotation, differences in processing between dorsal and palmar views) in an overall sample of 100 participants. As our protocol will strictly follow the same procedure, we do not provide specific pilot data in this regard.

Supplementary Information

Data analysis plan

<u>Data pre-processing:</u>

- TMS: For the EMG data collected in the neurophysiological assessment, raw EMG traces will be imported directly into R using the 'pyCFS' library[16] (originally Python-based, loaded within R). The raw signal will be offline bandpass filtered (20Hz-500Hz) and Notch-filtered (50Hz). The following processing steps will be carried out sequentially:
    1. Participant rejection: Participants will be excluded if they miss more than 2 out of 6 attention checks.
    2. Trial rejection: Trials where the root mean square (rms) of the filtered signal is >10µV in the 100ms before the first TMS artifact, illustrating possible pre-contraction of the muscle, will be rejected. Trials where coil position can be considered 'off target', according to the neuronavigation system, will be rejected. Although we will aim to maintain coil position below 3 units of deviation in all three parameters, we acknowledge that the expected accuracy can be slightly lower. Hence, we will reject trials where a deviation of >5mm in distance to target or >5° in tilt or rotation deviations is recorded. A participant will be excluded from the analysis if <50% of trials per experimental condition are available after the two trial removal steps.
    3. MEP collection: MEP amplitudes will be then extracted as the peak-to-peak amplitude of the filtered EMG signal for each muscle. Only the signal after the Test Stimulus (TS) artifact will be considered for this calculation. Previous studies suggest MEPs are unlikely to be observed at a latency below 18ms or above 50ms.[17] Therefore, we will consider the signal between 10ms and 50ms after the artifact in the calculation, to ensure that any MEPs produced with lower latencies are not missed. This process will be repeated for each trial and muscle in each participant.
    4. Corticospinal excitability: MEPs collected during the single-pulse TMS protocol, will be z-scored for each participant and muscle separately, to account for inter-individual and inter-muscle variability. This will make MEP changes comparable across participants, muscles and imagery conditions, and will be the dependent variable for the group-level analysis.
    5. Intracortical inhibition: For each condition, the average % of inhibition comparing the amplitude of the average conditioned MEP (paired-pulse TMS protocol) with the average unconditioned MEP (single-pulse TMS protocol), for each muscle independently, will be obtained. Formula: % Inhibition (%INH) = $(MEP_{conditioned}/MEP_{unconditioned})*100$. This will make inhibition comparable across participants, muscles and imagery and rest conditions. In this measure [0, ∞], smaller values indicate inhibition and values higher than 100 indicate facilitation. To optimise power in this analysis, each data point from the paired-pulse protocol will be normalised by the grand average of its single-pulse protocol, and then trial-to-trial data will be used for statistical analyses.
- MIQ-RS: Item-level responses will be collected and summed to obtain visual and kinesthetic subscale sum-scores. Participants will be excluded if they miss the attention check.
- CRFT: The execution time and imagery time (time elapsed between the space key press corresponding to the home circle and the next grey target), will be collected (i.e., 5 times per trial). An outlier analysis will be carried out following a top-down approach from participant to

trial, focusing on lower levels to detect errors such as double space bar presses.[14] Participants will be excluded if less than 50% of reaching movements are available for execution and imagery conditions after this analysis.

- HLJT: Reaction time will be collected and analysed. First, trials where reaction time is <300ms or >3,000ms, likely reflecting anticipatory responses and no engagement with the task, respectively, will be discarded.[15] For reaction time, the average time for each unique stimulus will be considered only for the trials with correct responses. The measure of the biomechanical constraints effect will be the difference between the mean reaction time of medially vs. laterally rotated stimuli. We will exclude all participants who have: 1) <60% overall accuracy, which is considered as responding at close to chance level; or 2) >50% trials rejected due to short or long reaction times.

<u>Manipulation and methodological checks:</u>

For simplicity and because we expect moderate-to-large effect sizes in the following analyses, frequentist NHST will be employed. Type I error rate will be set at 5%. Corrections for multiple comparisons will be applied by Bonferroni's procedure where appropriate. Linear mixed-effects models with by-participant random intercepts and slopes will be used throughout, using the 'lmerTest' package v3.1-3. If mixed models with full random structure show convergence or singularity problems, simpler models will be used by dropping factors from the random structure in a sequential manner.

- TMS experiment: As in the Pilot data, we will first formally check a group-level increase in MEP amplitudes of the 'active' muscle in the movement imagery condition compared to the 'non-active muscle' and rest, in single-pulse TMS protocol trials only. For that we will run a model with z-scored MEP amplitude as the dependent variable and condition (3 levels: 'active' imagery, 'non-active' imagery and rest) as predictor (MEP_{sp} ~ Condition + (1 + Condition|Participant)). We will also check the effect of intracortical 'disinhibition' of movement imagery, where an effect of condition on the %INH will be observed, whereby inhibition would be higher in the 'non-active' imagery and rest conditions compared to the 'active' imagery condition (%INH ~ Condition + (1 + Condition|Participant)).
- MIQ-RS: we will remove any participants that fail the attention check question. We will not perform any specific manipulation or methodological analyses.
- CRFT: we will assess the fundamental expected effect of index of difficulty (ID) on both execution and imagery times. Therefore, we will assess whether Reach Time increases linearly with ID for both conditions by a formal generalised mixed model with a Gamma distribution, as movement times are typically right-skewed (Reach Time ~ ID*Condition + (1 + ID*Condition|Participant)). We expect a moderate-to-large effect (slope > 0 and approximately linear for both imagery and execution). As a secondary measure, we will also check differences in y-intercepts between execution and imagery.
- HLJT: we expect a strong effect of Rotation Angle on reaction time. We also expect the presence of the biomechanical constraints effect for reaction time, in the palmar view only, therefore an interaction between Angle and View. To confirm this, we will run two models (Reaction time ~ Angle*View + (1 + Angle*View|Participant) for general effects; Reaction time ~ Direction*View + (1+Direction*View|Participant) for the biomechanical constraints).

Power Analyses

A 'Bayesian Power analysis' has been conducted using simulation. We have simulated two bivariate normal distributions that are correlated with small, moderate and large correlation coefficients (from r = 0.1 to r = 0.9 in steps of 0.1, i.e., alternative hypotheses or H1 with different effect sizes), or no correlation (r = 0, i.e., null hypothesis or H0). We have performed Bayesian correlations over these simulated scenarios and obtained the Bayes Factor (BF) of each simulation. The process has been repeated 1,000 times. Then, we have calculated the proportion of simulations that show strong evidence in favour of H1 (BF_{10} > 10), moderate evidence in favour of H0 (BF_{01} > 3), or no evidence in favour of neither hypothesis (BF_{01} > 3 and BF_{10} < 10) for each effect size. This can be considered the equivalent to statistical 'Power' in the Bayesian framework. We have carried out this analysis for each expected sample size (data look) depending on our frequentist-based sample size calculations (from N=70 participants to a maximum N=140, with increments of N=10 participants). The prior has been shrunk to control for multiple comparisons (see below for details).

The results are shown in Supplementary Figure 4. For a true null hypothesis (r = 0), the Bayesian 'Power' would be 68% or greater to obtain moderate evidence in favour of H0 across sample sizes. The false positive rate (strong evidence in favour of H1) would be consistently <1%, and no evidence for neither hypothesis would occur 20-30% of time. For a true alternative hypothesis with a small effect size (r = 0.3), the false negative rate (moderate evidence in favour of H0) would be <10% and would decrease as sample size increases. Similarly, the true positive rate (strong evidence in favour of H1) would increase as sample size increases, reaching 77% 'Power' at N = 140. The proportion of evidence for neither hypothesis would be 55% at N = 70 and would decrease as sample size increases. For larger effect sizes (r = 0.4), 'Power' would be >80% at N = 90, with false negative rates <1%. For even larger effect sizes (r ≥ 0.5), the 'Power' would be >80% at N = 70. Inconclusive evidence will be primarily obtained for marginal effect sizes (r = 0.1 and r = 0.2) and would reduce as effect size and sample size increase.

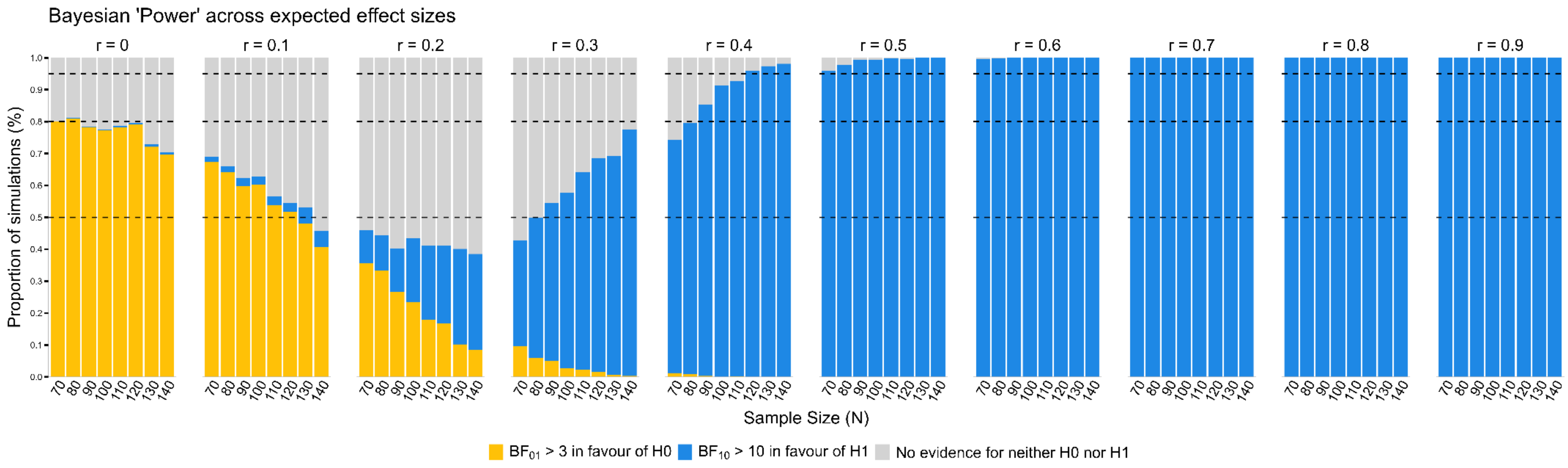

Supplementary Figure 4. *Bayesian 'Power' curves for each sample size and expected effect sizes. Horizontal dashed lines show reference for 50%, 80% and 95% 'power'. Sample sizes are only shown from N=70 to N=140 in steps of N=10 based on the frequentist sample size calculations and stopping criterion approach. Power is based on a two-tailed Pearson's correlation coefficient.*

Prior shrinkage for sequential analyses

Bayesian analyses which use Bayes Factors to make dichotomous decisions on effect existence or significance (i.e. presence/absence of an effect) require error control.[18] However, there is no straightforward method or consensus on how to achieve it in the Bayesian framework (i.e. no Bonferroni correction-like procedures are available). We will use dynamic prior specification with a linear interpolation as a way to control for multiple comparisons due to the optional stopping approach. The default prior distribution for the null hypothesis for a correlation coefficient is a standard Cauchy distribution with limits [-1, +1], location = 0 and a rscale parameter which controls the spread of the distribution (Supplementary Figure 5A).[19] Higher values of rscale make the distribution flatter, thus allowing extreme values of the correlation coefficient to be plausible (Fig. S4A). Lower values make the distribution approximate a t-student distribution with heavy tails, where most of the probability density is around the location, thus making extreme values less plausible yet still possible. The default value of 0.707 can be considered a wide prior which allows large values of the correlation coefficient to be completely plausible, and values of rscale = 1 are considered a very wide prior. Therefore, we will start our first data look (N = 70 participants) at rscale = 1, reflecting a very weak expectation. Afterwards, the rscale value will be decreased in a linear fashion (Supplementary Figure 5B) every time 10 new participants are enrolled, so that by the maximum sample size (N = 140 participants), rscale = 0.3. This could be considered a very skeptical prior which places most of the probability density near the location (i.e., correlation coefficient = 0 or null hypothesis). Following this approach, every data look the prior will be dynamically adjusted (N = 80, rscale = 0.9; N = 90, rscale = 0.8; N = 100, rscale = 0.7; N = 110, rscale = 0.6, N = 120, rscale = 0.5, N = 130, rscale = 0.4, N = 140, rscale = 0.3; Supplementary Figure 5C). This prior shrinkage will effectively adjust the false positive rate if the null hypothesis is true. Note this method *does not* adjust the false negative rate if the alternative hypothesis is true. Because we are more concerned about false positives than false negatives, we think this approach is valuable in our case.

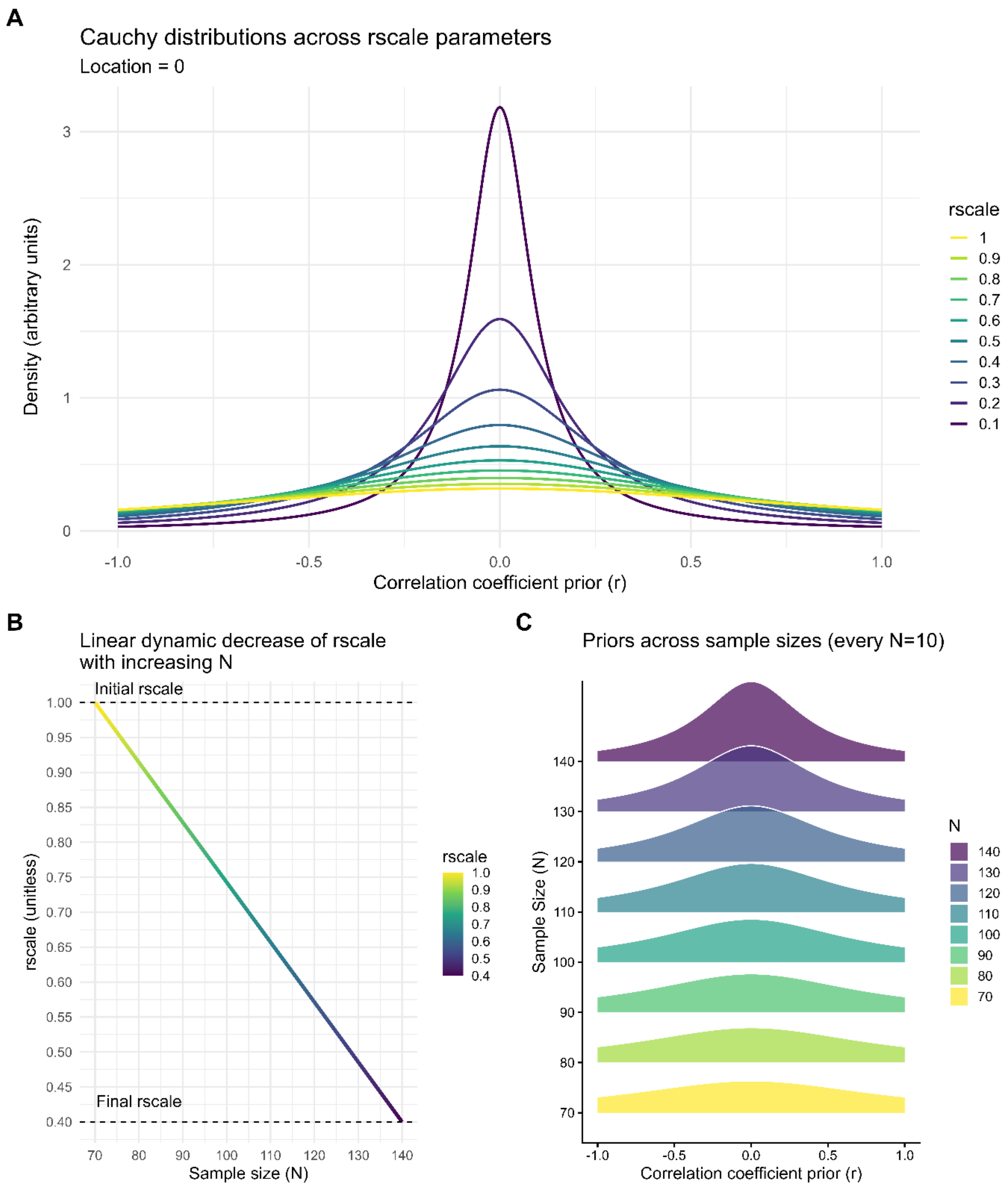

Supplementary Figure 5. *Dynamic prior specification.* ***Panel A*** *shows possible distributions for the correlation coefficient prior according to the rscale parameter values (density is unitless, the height of the curve at any given x shows how dense the data are around that x value; the area under the curve between two x values gives the probability of the variable falling within that range). The panel shows how decreasing the rscale parameter makes the distribution more concentrated around 0, indicating stronger skepticism.* ***Panel B*** *shows the decrease of the rscale parameter (unitless) as a linear interpolation of the sample size, in order to make the prior more skeptical as the number of data looks increases.* ***Panel C*** *shows the final distributions for the null hypothesis according to the corresponding data look.*

References

1. Neige C, Monany DR, Stinear CM, Byblow WD, Papaxanthis C, Lebon F. Unravelling the Modulation of Intracortical Inhibition During Motor Imagery: An Adaptive Threshold-Hunting Study. *Neuroscience*. 2020;434:102-110. doi:10.1016/j.neuroscience.2020.03.038
2. Kumru H, Soto O, Casanova J, Valls-Sole J. Motor cortex excitability changes during imagery of simple reaction time. *Exp Brain Res*. 2008;189(3):373-378. doi:10.1007/s00221-008-1433-6
3. Liepert J, Neveling N. Motor excitability during imagination and observation of foot dorsiflexions. *J Neural Transm Vienna Austria 1996*. 2009;116(12):1613-1619. doi:10.1007/s00702-009-0287-9
4. Abbruzzese G, Assini A, Buccolieri A, Marchese R, Trompetto C. Changes of intracortical inhibition during motor imagery in human subjects. *Neurosci Lett*. 1999;263(2-3):113-116. doi:10.1016/s0304-3940(99)00120-2
5. Stinear CM, Byblow WD. Role of intracortical inhibition in selective hand muscle activation. *J Neurophysiol*. 2003;89(4):2014-2020. doi:10.1152/jn.00925.2002
6. Fadiga L, Buccino G, Craighero L, Fogassi L, Gallese V, Pavesi G. Corticospinal excitability is specifically modulated by motor imagery: a magnetic stimulation study. *Neuropsychologia*. 1999;37(2):147-158. doi:10.1016/s0028-3932(98)00089-x
7. Lebon F, Byblow WD, Collet C, Guillot A, Stinear CM. The modulation of motor cortex excitability during motor imagery depends on imagery quality. *Eur J Neurosci*. 2012;35(2):323-331. doi:10.1111/j.1460-9568.2011.07938.x
8. Stinear CM, Byblow WD. Modulation of corticospinal excitability and intracortical inhibition during motor imagery is task-dependent. *Exp Brain Res*. 2004;157(3):351-358. doi:10.1007/s00221-004-1851-z
9. Neige C, Ciechelski V, Lebon F. The recruitment of indirect waves within primary motor cortex during motor imagery: A directional transcranial magnetic stimulation study. *Eur J Neurosci*. 2022;56(12):6187-6200. doi:10.1111/ejn.15843
10. Sohn YH, Dang N, Hallett M. Suppression of Corticospinal Excitability During Negative Motor Imagery. *J Neurophysiol*. 2003;90(4):2303-2309. doi:10.1152/jn.00206.2003
11. Ridding MC, Rothwell JC. Afferent input and cortical organisation: a study with magnetic stimulation. *Exp Brain Res*. 1999;126(4):536-544. doi:10.1007/s002210050762
12. Gregg M, Hall C, Butler A. The MIQ-RS: A suitable Option for examining movement imagery ability. *Evid Based Complement Alternat Med*. 2007;7(2):249-257. doi:10.1093/ecam/nem170
13. Fitts PM. The information capacity of the human motor system in controlling the amplitude of movement. *J Exp Psychol*. 1954;47(6):381-391. doi:10.1037/h0055392
14. Czilczer C, Sachse P, Dahm S. One size Fitts' all? Reconsidering the use of deviations from real-time action imagery in mental chronometry tasks. *OSF*. Preprint posted online June 23, 2025. doi:10.31234/osf.io/c2mw6_v1
15. Moreno-Verdú M, McAteer SM, Waltzing BM, Caenegem EV, Hardwick RM. Development and validation of an open-source Hand Laterality Judgement Task for in-person and online studies. *bioRxiv*. Preprint posted online October 21, 2024:2024.10.17.618819. doi:10.1101/2024.10.17.618819
16. Hurst A. a-hurst/pycfs-signal. Published online April 4, 2024. Accessed January 29, 2025. https://github.com/a-hurst/pycfs-signal
17. Šoda J, Vidaković MR, Lorincz J, Jerković A, Vujović I. A Novel Latency Estimation Algorithm of Motor Evoked Potential Signals. *IEEE Access*. 2020;8:193356-193374. doi:10.1109/ACCESS.2020.3033075
18. Lakens D. *Improving Your Statistical Inferences*. Zenodo; 2022. doi:10.5281/ZENODO.6409077
19. Makowski D, Ben-Shachar MS, Patil I, Lüdecke D. Methods and Algorithms for Correlation Analysis in R. *J Open Source Softw*. 2020;5(51):2306. doi:10.21105/joss.02306

Qualitative reports (self-assessments) during imagery

All questions will be formulated to be able to answer them via 11-point rating scales (from 0 to 10), unless otherwise indicated.

TMS experiment:

1. Open-ended question: Please, describe the content of the imagery you performed.
2. What was your use of the visual modality of imagery (creating a visual image of the movement)?
3. If you used visual imagery, what was the use a first-person perspective (watching the movement through the eyes of the performer, from an *internal* point of view)?
4. If you used visual imagery, what was the use a third-person perspective (watching yourself performing the movement, from an *external* point of view)?
5. What was your use of the kinesthetic modality of imagery (feeling the sensations of the movement)?
6. What was your ability to *generate* movement imagery (see/feel your hand and fingers and their movement)?
7. What was your ability to *maintain* movement imagery throughout the required period (maintaining the contraction from appearance of the green circle until it disappeared)?
8. What was your ability to *control* movement imagery throughout the required period (controlling the change in position of your finger)?
9. To what extent where you able to isolate movement imagery (imagining moving only the finger requested, and not another finger or body part)?
10. Did you imagine your entire hand, or were you able to isolate imagery to the required finger (index or little where requested)?
11. Did you have any preference in terms of fingers during imagery (would you say imagery was easier overall for one finger compared to the other)?

MIQ-RS:

1. What is your preference for using a particular imagery modality (from totally visual to totally kinesthetic)?
2. What perspective did you take while completing the movements in the visual modality (from totally first-person to totally third-person)?
3. When completing the visual modality items, did you switch to kinesthetic imagery?
4. When completing the kinesthetic modality items, did you switch to visual imagery?

CRFT:

1. What was your use of the visual modality of imagery (creating a visual image of the movement)?
2. If you used visual imagery, what was the use a first-person perspective (watching the movement through the eyes of the performer, from an *internal* point of view)?
3. If you used visual imagery, what was the use a third-person perspective (watching yourself performing the movement, from an *external* point of view)?
4. What was your use of the kinesthetic modality of imagery (feeling the sensations of the movement)?
5. What was the vividness of the visual modality of imagery (considering the clarity of the image)?

6. What was the vividness of the kinesthetic modality of imagery (considering the intensity of the sensations)?
7. What was the vividness of the tactile modality of imagery (the texture of the objects)?
8. What was the vividness of the auditory modality of imagery (the hearing of sounds)?
9. What was your ability to *generate* movement imagery (see/feel your hand and fingers and their movement)?
10. What was your ability to *maintain* movement imagery throughout the required period (from first tap to last tap)?
11. What was your ability to *control* movement imagery throughout the required period (controlling the change in position of your arm and hand throughout the taps)?
12. How quickly was imagery created?
13. Did you imagine yourself making errors (e.g., not hitting on the target)?
14. On average, how long was imagery held?

HLJT:

1. Open-ended question: Please, describe which strategy or strategies (if any) did you use to solve the task. If you did not use any specific strategy, please write "I did not use any strategy".
2. Yes/No question: In the task, were you thinking about your own hands to decide the laterality?
3. Follow-up, open-ended question from the previous question: Please briefly explain.
4. Yes/No question: In the task, did you imagine moving your hands to make the laterality judgements?
5. Follow-up, open-ended question from the previous question: Please briefly explain.
6. If you used imagery during the task, which hand were you imagining? (Options: left, right, both).
7. What was your use of the visual modality of imagery (creating a visual image of the movement)?
8. If you used visual imagery, what was the use a first-person perspective (watching the movement through the eyes of the performer, from an *internal* point of view)?
9. If you used visual imagery, what was the use a third-person perspective (watching yourself performing the movement, from an *external* point of view)?
10. What was your use of the kinesthetic modality of imagery (feeling the sensations of the movement)?
